# Enhancement of Immune Response and Anti-Infection of Mice by Porcine Antimicrobial Peptides and Interleukin-4/6 Fusion Gene Encapsulated in Chitosan Nanoparticles

**DOI:** 10.3390/vaccines8030552

**Published:** 2020-09-21

**Authors:** Junjie Peng, Yongle Xiao, Xiaoping Wan, Qian Chen, Huan Wang, Jiangling Li, Jianlin Chen, Rong Gao

**Affiliations:** 1 College of Life Science, Sichuan University, Chengdu 610065, China; pengjunjie93@163.com (J.P.); xiao_yongle@163.com (Y.X.); xiaopingwan125@gmail.com (X.W.); cqcqcyy@163.com (Q.C.); qmyy128481@163.com (H.W.); 2 Sichuan Animal Science Academy, Chengdu 610066, China; yujiang1465@126.com; 3 School of Laboratory Medicine, Chengdu Medical College, Chengdu 610500, China

**Keywords:** cathelicidin antimicrobial peptides, fusion porcine interleukin-4/6, chitosan nanoparticles, mice, resistance, challenge

## Abstract

In order to develop a novel and effective immunoregulator to enhance both the immune response and antimicrobial function, a recombinant eukaryotic expression plasmid-pVAX1 co-expressing fusion cathelicidin antimicrobial peptides (CAMPs) and fusion porcine interleukin-4/6 gene (IL-4/6) was constructed and encapsulated in chitosan nanoparticles (CS-VAP4/6), prepared by the ionotropic gelation method. Four-week-old female Kunming mice were divided into three groups and intramuscularly injected, respectively, with CS-VAP, CS-VAP4/6, and CS-pVAX1. On 28 days post-inoculation, the mice were challenged by intraperitoneal injection with *Staphylococcus aureus* (ATCC 25923) and *Escherichia coli* (ATCC 25922); IgG, IgG1 and IgG2a, CD4+, and CD8+ T cells increased significantly in the VAP- and VAP4/6- treated mice, detected by ELISA and flow cytometry, correspondingly (*p <* 0.05). As analyzed by qPCR, expression levels of Toll-like receptor (TLR) 1, TLR4, TLR6, TLR9, IL-1, IL-2, IL-4, IL-6, IL-7, IL-12, IL-15, IL-23, Tumor Necrosis Factor (TNF)-α, and Interferon-gamma (IFN-γ) genes were also significantly up-regulated in comparison with those of the control mice (*p <* 0.05). Their immunological markers were elevated significantly to different degrees in CS-VAP4/6-treated mice compared with CS-VAP in different days post-inoculation (*p <* 0.05). After challenge with *E. coli* and *Staphylococcus aureus*, most of the VAP- and VAP4/6- treated mice survived, and no symptoms of bacterial infection were observed. In contrast, 80% of control mice died of infection. Among the treated groups, VAP4/6 had a stronger resistance against challenge with *E. coli* infection. These results demonstrated that the fusion gene of antimicrobial peptide and interleukin-4/6 has the promising potential as a safe and effective immunomodulator for the control of bacterial infections.

## 1. Introduction

Drug resistance of animal pathogens is a continuously growing problem, and immunosuppressive pathogens worsen mixed infections to animals, which is still a massive challenge for the prevention and control of animal infectious diseases. Moreover, antibiotics have been banned as growth promoters in 86 countries and regions around the world since 2018, and all varieties of growth-promoting antimicrobial additives will be prohibited by the end of 2020 in China. Therefore, there is a pressing need for the replacement of antimicrobial additives to prevent complicated infection of animals, as well as to improve their innate and acquired immunity, particularly to strengthen the animal mucosal immune defense responses and antibacterial ability. In order to decrease the occurrence of drug resistance, more effective roles of T helper cells in optimizing antibody immune response and effector CD4^+^ or CD8^+^ T cells in eliminating intracellular pathogens are greatly expected [1]. 

Cytokines have been predicted to be much milder and far more efficient adjuvants [2]. Relevant evaluations of the immune effect of cytokines as vaccine adjuvants have been conducted [3,4]. Interleukin-4 (IL-4) is an essential immunomodulator, which can stimulate the expression of immunoglobulins selectively. Interleukin-6 (IL-6) is one of the multiple functional cytokines with the ability to promote a variety of cell proliferation and differentiation. Both IL-4 and IL-6 are canonical type 2 cytokines, which are known to have the ability to significantly promote the proliferation of B lymphocyte cell and induce the type 2 immune response. 

Antimicrobial peptides have been recognized as a vital component of the host due to their strong antibiotic activity in a wide range of microorganisms including bacteria, fungi, and enveloped viruses. Otherwise, they can also trigger a specific defense of the host [5]. With the ability to modulate the innate immune response of the host, cathelicidin antimicrobial peptides (CAMPs) became a focus of adjuvants. Proline-arginine-rich 39-amino acid peptide (PR-39) is a proline-rich peptide, which carries out antibacterial function by terminating protein and DNA synthesis [6]. Other biological functions such as prevention of inflammation of the tissue injury, enhancement of the wound repair ability, and regulation of angiogenesis have been reported [7]. Tritrpticin, a member of cathelicidin family, has a broad spectrum of antibiotic activity. It acts through non-specific binding to the cytoplasmic membrane instead of binding to a specific protein receptor [8]. Therefore, as more and more drug-resistant strains emerge, tritrpticin still shows a lower rate of inducing drug resistance. Porcine myeloid antimicrobial peptide-23 (PMAP-23) belongs to the amphipathic α-helical peptides group, which was reported to show potent antimicrobial activity against Gram-negative and Gram-positive bacteria without hemolytic activity [9]. Protegrin-1(PG-1), a cysteine-rich β-hairpin antimicrobial peptide with 18 residues, was found out to be a potent antimicrobial agent with in vivo activity against clinically relevant, antibiotic-resistant bacteria [10] (Table 1).

Our previous studies demonstrated that IL-4, IL6, and fusion IL-4/6 genes can significantly enhance the immunity of mice or piglets [11]. In order to verify the potential of fusion CAMPs and cytokines as immunomodulators, fusion gene of CAMPs and IL-4/IL-6 were constructed in this research to evaluate its effects on the immune response of mice. The chitosan was employed as the packing material due to its remarkable property of plasmid DNA delivery [12,13].

## 2. Materials and Methods

### 2.1. Construction of the Recombinant Eukaryotic Expression Plasmid

Plasmid pVAX1 (Invitrogen Company, Carlsbad, CA, USA) was used as the eukaryotic expression vector of DNA vaccine. Endo-Free Plasmid Giga Kit (OMEGA) was employed to extract plasmids among the experiment. The fusion gene of IL-4 and IL-6 (IL-4/6) was constructed previously in our lab by inserting the IL-4/6 fusion gene into the expression VR1020 plasmid (Genbank AY 294020 and AF 493992).

The recombinant gene (TPA-PR39-linker-Tritrpticin-linker-PAMP23-linker-Protegrin1-His-tag) of downstream tissue plasminogen activator (TPA) signal sequence and antimicrobial peptides fusion gene-CAMPs, which was connected by GSGDDDDK linker with each other, was amplified from pVR1020-CAMPs (VRP), which constructed in our lab previously. PCR primers for the TPA-CAMPs sequences were P1:5′-GTTTAAACTTAAGCTTATGGATGCAATGAAGAGAG-3′ and P2:5′-AAACGGGCCCTCTAGACTAATGGTGATGGTGATGATGCT-3′. Then, TPA-CAMPs were subcloned into pVAX1 to construct recombinant plasmid pVAX1-CAMPs (designated as VAP) using the In-fusion cloning technique. Furthermore, linkage of IL-4/6 and TPA-CAMPs was achieved by the use of the 63 bp 2A self-cleaving sequence (GGA AGC GGA GAG GGC AGG GGA AGT CTT CTA ACA TGC GGG GAC GTG GAG GAA AAT CCC GGG CCA) to make sure that the proteins are expressed singly. The fused fragment was subcloned into the eukaryotic expression plasmid pVAX1 (designated as VAP4/6).

### 2.2. Large-Scale Preparation of VAP, VAP4/6, and pVAX1

VAP, VAP4/6, and pVAX1 were transformed into *Escherichia coli* (*E. coli*) DH5α competent cells (Transgene Biotech, Beijing, China), respectively, and cultured in the Lurea Bertani (LB) medium with 100 µg/mL kanamycin (Kana) at 37 °C for 12 h. The positive clones were confirmed by colony PCR assay and inoculated in LB broth with kana (100 µg/mL) at 37 °C and shaken at 200 rpm for 15 h. Bacterial cells were pelleted by centrifugation and plasmid DNA were extracted by large-scale alkine lysis. Then spermine method was used to precipitate and purify extracted plasmids [14]. Residual contamination by endogenous toxin of *E. coli* was measured by Limulus amebocyte lysate test to be within 0.1 EU/mg [15]. Subsequently, the plasmids were resuspended in sterile water and stored at −20 °C until use.

### 2.3. Preparation and Detection of Recombinant Plasmids Encapsulated in Chitosan Nanoparticles

Chitosan (CS) was provided by Sigma Aldrich (St. Louis, MO, USA). Its molecular weight (MW) was 150 kDa with 95% deacetylation. Plasmid VAP, VAP4/6, and pVAX1 entrapped in CS (designated as CS-VAP, CS-VAP4/6, and CS-pVAX1, respectively) were prepared by the ionotropic gelation method [16]. Briefly, CS was dissolved in 1% glacial acetic acid buffer (pH 5.5) to a concentration of 2.4 mg/mL and filtered through 0.22 µm Millipore filter to remove bacteria. Then each recombinant plasmid was incubated in 55 °C for 20 min mixed with sodium polyphosphate solution. At last, each plasmid solution was gently dripped into CS solution until mass ratio (CS to plasmid) reached 30:1 in 50–55 °C water bath with magnetic stirring. Then, the solution was incubated for 10 min to form CS-recombinant plasmid. The granule diameter, dispersion rate, and zeta electronic potential were measured by a Zetasizer3000 HS/IHPL instrument (Malvern Instruments Ltd., Malvern, UK).

### 2.4. Biological Activity Assay of CS-VAP, CS-VAP4/6, and CS-pVAX1 in Pig Lymphocytes

HEK 293 cells (1.0 × 10^5^ cells/well) transfected with CS-VAP, CS-VAP4/6, and CS-pVAX1 (4 µg plasmid DNA/well), respectively, were to express CAMPs and IL-4/6 protein. Green fluorescent protein (GFP) plasmid was used as a positive control. The transfected cells were harvested at 24, 48, and 72 h, respectively. Reverse transcription polymerase chain reaction (RT-PCR) was employed to assess mRNA level. The cell supernatant from three time points was used to stimulate the lymphocyte proliferation through Counting Kit-8 (CCK8 Yiyuan biotechnology, Guangzhou, China). Pig peripheral blood mononuclear immune (PBMI) cells separated by Lymphocyte Separation Medium (LSM, ficoll 400) were diluted to 2 × 10^6^ cells per milliliter and cultured in the dishes for 10 mL, then the pig lymphoblast was incubated for 24 h at 37 °C, and 5% CO_2_ stimulated with 5 µg/mL Con A (Sigma Chemical Co., St. Louis, MO, USA). The RPMI 1640 medium was used to modulate the cell concentration to 6 × 10^6^ cells per milliliter, then 50 µL cells were incubated with 50 µL sample supernatants at 37 °C and 5% CO_2_. Each sample was divided into three duplicate wells, and RPMI 1640, PBS, and 100 µL pig lymphoblast cells were set as the control group. CCK8 (10 µL) was added to each well at 48 h. After incubating for another 2 h, the absorbances at 450 nm of each sample were determined, using a microplate reader 3550 (Bio-Rad, Hercules, CA, USA).

### 2.5. Animal Vaccination

Thirty healthy 4-week female Kunming mice (purchased from the Animal Center of West China Center of Medical Sciences, Sichuan University, Chengdu, China) were assigned to 3 groups (CS-VAP, CS-VAP4/6, and CS-pVAX1) randomly. Each group was injected intraperitoneally (IP) with 0.2 mL recombinant plasmid (0.5 mg/mL), respectively. Group CS-pVAX1 was set as a negative control. Peripheral blood samples were collected weekly from the tail vein of mice on weeks 0, 1, 2, 3, 4 after injection to evaluate the immunological changes. Finally, *Staphylococcus aureus* (ATCC 25923) and *E. coli* (ATCC 25922) stored in our laboratory were used to challenge the mice at 28 days. The bacteria were cultured in LB culture. Bacteria broth culture (0.2 mL) (10^9^ CFU/mL) corresponding to 10^7^ CFU/g of body weight was injected into the mice, respectively. Each bacterium was intraperitoneally injected into 5 mice. All mice were fed under the same conditions. The care and use of experimental animals complied with Chinese animal welfare laws, guidelines, and regulations (SYXK-Chuan-2018-185).

### 2.6. Immunological Assays In Vivo

#### 2.6.1. Assay of CD4 and CD8 Positive T Cells by Flow Cytometry

Anti-Mouse CD8a and Anti-Mouse CD4, labelled with phycoerythrin (PE) and PerCP-Cy5.5, respectively, were purchased from eBioscience (San Diego, CA, USA) Each test contained 50 µL of peripheral blood sample, and then incubated 0.25 µL PE labeled Anti-Mouse CD8a and 0.25 µL PerCP-Cy5.5 labeled Anti-Mouse CD4. After incubating for 20 min in the dark, 2 mL lysing solution (Becton Dickinson, Franklin Lakes, NJ, USA, 10%, v/v) was added to make the lysis of erythrocytes more complete and surviving cells were washed twice with PBS, with centrifugation between each step for 5 min at 500 g. FACScan flow cytometer was used for FCM analysis (BD Biosciences, San Jose, CA, USA). First, an electronic gate was set on lymphocytes and then gated CD4+ T cells in Y axis and CD8+ cells as figure. Dead cells were excluded according to the forward scatter and side scatter.

#### 2.6.2. Assays of IgG, IgG1, IgG2 by Sandwich ELISA

Mouse Immunoglobin G (IgG), Immunoglobin G1 (IgG1), and Immunoglobin G2a (IgG2a) quantitation ELISA Kits (ebioscience, San Diego, CA, USA) were employed to detect the changes of immunoglobulins in sera. The sandwich ELISA was conducted according to the manufacturer’s protocols. In brief, adequate amounts of mouse serum samples were transferred to a 96-well plate coated with capture antibody. The plate was incubated for 1 h and applied with secondary antibody conjugated with HRP for more specific binding. After three times washing, tetramethyl benzidine (TMB) was added for target immunoglobin detection. Optical density (OD) were measured at 450 nm in microplate reader 3550 (Bia-rad, Hercules, CA, USA).

#### 2.6.3. Analysis of Immune Gene Expression by Quantitative Real-Time Polymerase Chain Reaction (QRT-PCR)

RNAiso Plus (1 mL) (TaKaRa Bio, Kusatsu, Japan) was added into blood samples to get RNA extract. Total RNA extract was reverse-transcribed at 42 °C for 15 min using TransGen., TransScript All-in-one First-Strand cDNA Synthesis Super Mix for qPCR (One-step gDNA Removal) as described in the manufacturer’s guide. Primers were designed and synthesized according to the cDNA sequence of mouse β-actin, interleukin-1 (IL-1), interleukin-2 (IL-2), interleukin-4 (IL-4), interleukin-6 (IL-6), interleukin-7 (IL-7), interleukin-12 (IL-12), interleukin-15 (IL-15), interleukin-23 (IL-23), toll-like receptor 1 (TLR1), toll-like receptor 4 (TLR4), toll-like receptor 6 (TLR6), toll-like receptor 9 (TLR9), CAMP, interferon γ (IFN-γ), and tumor necrosis factor α (TNF-α), reported in GenBank (Table 2). 

Real-time PCR was performed on the iQ5 PCR detection system (Bio-Rad, Singapore) with an initial denaturation for 30 s at 95 °C, followed by 40 cycles of 5 s at 95 °C, 30 s at optimal annealing temperature, at the end of all cycles, a melt curve, from 65 °C to 95 °C per 6 s. Each RT-PCR reaction was carried out in a total volume of 15 µL, including 1 µL cDNA, 7.5 µL SsoAdvanceTM Universal SYBR Green Supermix (BioRad, Singapore), and 0.25 µL each of paired primers and 6 µL ddH_2_O. Beta-actin gene was used as the reference and the mRNA levels of immunity-related genes were calculated by the geometric means method and the formula: relative level = 2^−ΔΔCt^ [17].

### 2.7. Statistical Analysis

GraphPad Prism6 (Graphpad Software, San Diego, CA, USA) was used to manage and analyze the data. Comparisons between two groups were performed using the two-tailed Student’s *t*-test. *p <* 0.05 was set to be significant.

## 3. Results

### 3.1. Preparation of Recombinant Plasmids

**Figure 1:**
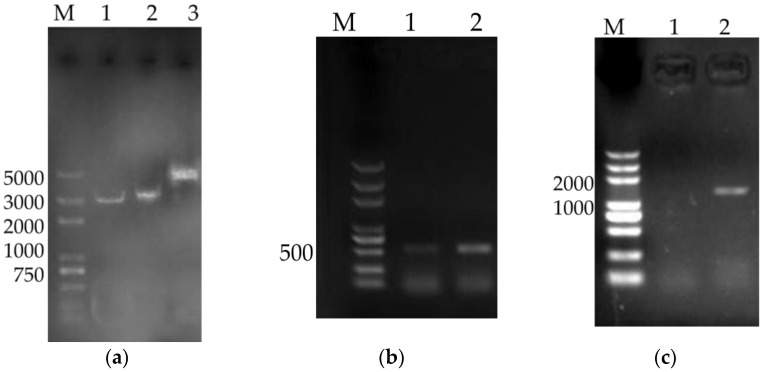
Agarose gel electrophoresis of VAP and VAP4/6 (**a**) (lane M: DL 5000 DNA Marker, lane 1: pVAX1 2999 bp, lane 2: VAP, lane 3: VAP4/6). Polymerase chain reaction (PCR) of cathelicidin antimicrobial peptides (CAMPs) (**b**) (lane M: DL 5000 DNA Marker, lane 1-2: VAP,) and CAMPs-IL-4/6 (**c**) (lane M: DL 5000 DNA Marker, lane 1: Negative Control, lane 2: VAP4/6).

Recombinant plasmids of VAP (3515 bp) and VAP4/6 (4406 bp) were extracted following large-scale alkine lysis and analyzed by agarose gel electrophoresis (Figure 1a). CAPMs (516 bp) and CAMPs-IL-4/6 (1407 bp) genes were amplified successfully by PCR and analyzed by agarose gel electrophoresis (Figure 1b,c).

### 3.2. Preparation of Chitosan Nanoparticles

**Figure 2:**
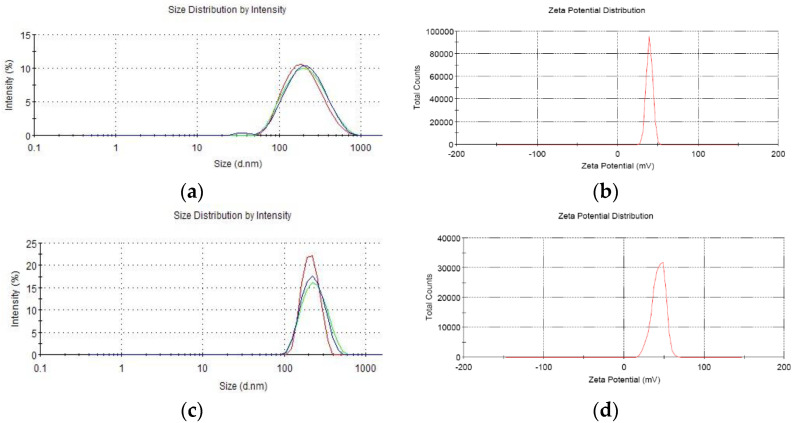

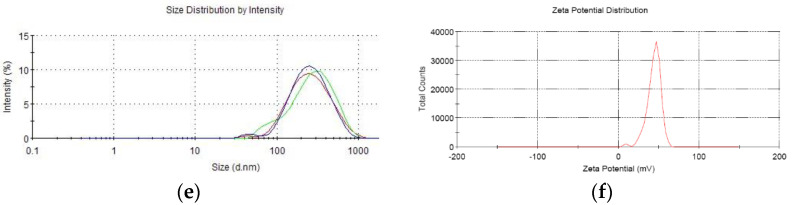
The parameters of average granule diameters and Zeta potentials of chitosan nanoparticles. (**a**,**c**,**e**) The average granule diameters of CS-VAP, CS-VAP4/6, and CS-pVAX1, respectively; Each color represents one test. (**b**,**d**,**f**) The Zeta potentials of CS-VAP, CS-VAP4/6, and CS-pVAX1, respectively.

Analysis by Zetasizer 3000 HS/IHPL showed that the average granule diameters of CS-VAP, CS-VAP4/6, and CS-pVAX1 were 292.12 ± 9.23 nm, 327.53 ± 8.17 nm, and 278.06 ± 7.55 nm, respectively, and the Zeta potentials were + 36.25 mV, + 35.31 mV, and + 38.39 mV (Figure 2), suggesting that the CS-VAP, CS-VAP4/6, and CS-pVAX1 were positively charged.

### 3.3. Bioactivity Assay of VAP and VAP4/6 Proteins In Vitro

**Figure 3:**
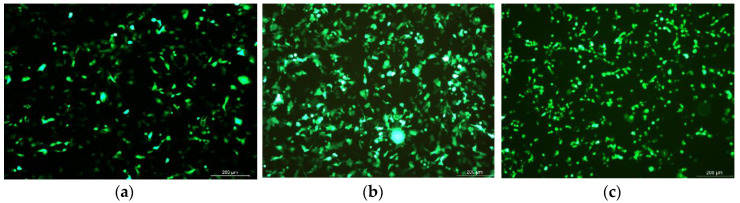
Green fluorescent protein (GFP) fluorescence protein expression in the HEK 293 cells ((**a**): 24 h, (**b**): 48 h, (**c**): 72 h).

**Figure 4:**
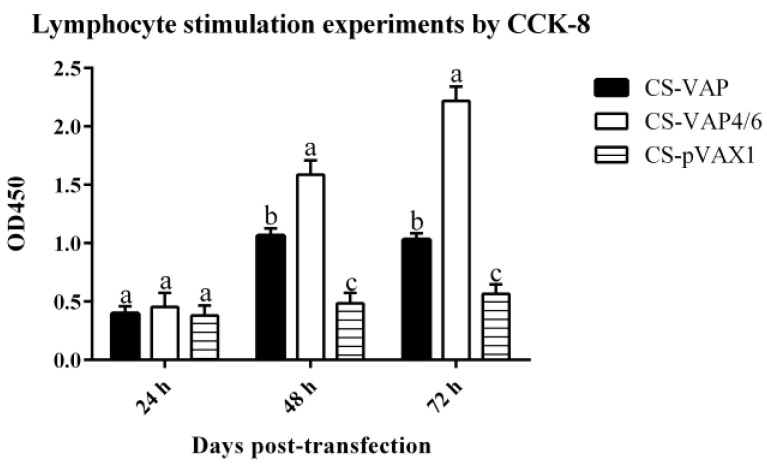
The proliferation results of pig lymphoblast. The proliferation of treated groups had significant proliferations compared with the control group (*p <* 0.05), and the proliferation of CS-VAP4/6 was higher than CS-VAP at 48 and 72 h (*p <* 0.05). Each datum is the average of three replicates. The followings are the same as here.

Green fluorescent protein (GFP) was successfully expressed in HEK293 cells and the highest expression level was found at 48 h (Figure 3). CCK 8 assay shows that proliferation of pig lymphoblast of the treated groups was significantly increased compared with the control group at 48 and 72 h post-stimulation (*p <* 0.05). Moreover, CS-VAP4/6 had a higher proliferation compared with CS-VAP during this period (*p <* 0.05) (Figure 4).

### 3.4. Changes of Th and Tc Cells

**Figure 5:**
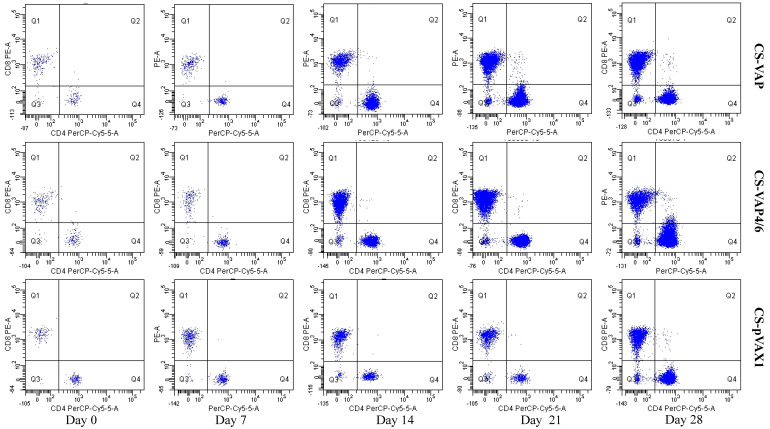
Changes of mice CD4+ and CD8+ T cells.

**Figure 6:**
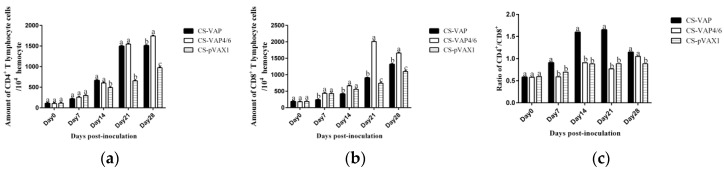
Changes of CD4+ (**a**) and CD8+ (**b**) T cells in the blood of experimental mice. (**c**) shows the ratio of CD4+ and CD8+ T cells. The amounts of CD4+ and CD8+ T cells significantly increased in the blood of treated groups at 21 and 28 days post-inoculation compared with the control group (*p <* 0.05). Moreover, the amounts of CD4+ and CD8+ T cells of CS-VAP4/6 rose significantly compared with CS-VAP at 28 days (*p <* 0.05). There was a significant increase in the ratio of CD4+ to CD8+ T cells of CS-VAP compared with CS-VAP4/6 and CS-pVAX1 from 7 to 21 days (*p <* 0.05).

CD4+ and CD8+ T lymphocyte cells of CS-VAP, CS-VAP4/6, and CS-pVAX1 groups in the peripheral blood were determined by FCM. As shown in Figure 5 and Figure 6a,b, the mounts of CD4+ and CD8+ cells increased significantly in CS-VAP and CS-VAP4/6 compared with CS-pVAX1 at 21 and 28 days (*p <* 0.05). Moreover, the amount of CD8+ cell of CS-VAP4/6 had a significant increase compared with CS-VAP at 21 and 28 days. Figure 6c shows that there was a significant increase in the ratio of CD4+ and CD8+ T cells of CS-VAP compared with CS-VAP4/6 and CS-pVAX1 from 7 to 21 days (*p <* 0.05), while there was no difference between CS-VAP4/6 and CS-pVAX1 at the same time (*p >* 0.05). At 28 days, the ratio of CD4+ and CD8+ T cells of treated groups increased significantly in comparison with the control (*p <* 0.05) (Figure 6c).

### 3.5. Changes of the Level of IgG, IgG1, and IgG2a

**Figure 7:**
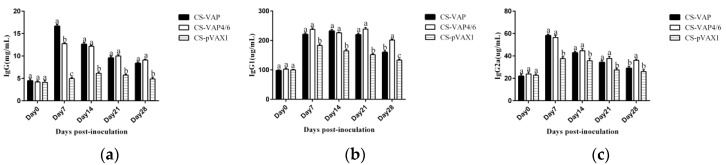
Changes of IgG (**a**), IgG1 (**b**), and IgG2a (**c**) levels of the experimental mice. The IgG, IgG1, and IgG2a levels of each treated group are higher than that of the control group from day 7 to day 28 post-inoculation.

The significant increases of IgG, IgG1, and IgG2a levels were found in sera of the treated groups (CS-VAP and CS-VAP4/6) compared with CS-pVAX1 from 7 days to 28 days post-inoculation (*p <* 0.05) (Figure 7a–c). At 28 days post-inoculation, the levels of IgG1 and IgG2a increased significantly in CS-VAP4/6 compared with CS-pVAX1 (*p <* 0.05) (Figure 7b,c).

### 3.6. Changes of Immune Gene Expression

#### 3.6.1. Changes of Expression Levels of TLR Genes

**Figure 8:**
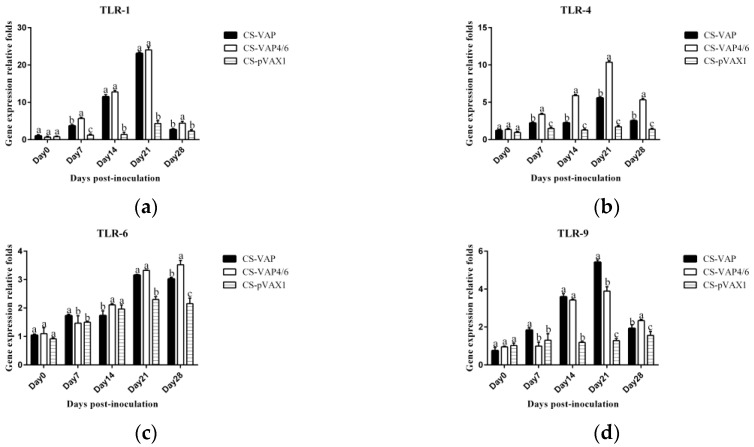
Changes of four toll-like receptor (TLR) genes in blood of experimental mice. The expression of TLR1 (**a**), TLR 4 (**b**), TLR 6 (**c**), and TLR9 (**d**) increased markedly in CS-VAP and CS-VAP4/6 in different days post-inoculation compared with CS-pVAX1 (*p <* 0.05), and the expressions of these four TLR genes of CS-VPA4/6 were promoted significantly in different periods after inoculation compared with CS-VAP(*p <* 0.05).

To evaluate the effect on mice innate immunity, the expression changes of four TLRs were detected. The results are shown in Figure 8. Significantly higher levels of TLR1 were seen in groups CS-VAP and CS-VAP4/6 from 7 to 21 days compared with CS-pVAX1 (*p <* 0.05); moreover, at 7 days, the expression level of TLR1 of CS-VAP4/6 increased significantly in comparison with CS-VAP (*p <* 0.05) (Figure 8a). Figure 8b shows that there were significant increases in the expression level of TLR4 of CS-VAP and CS-VAP4/6 compared with CS-pVAX1 from 7 to 28 days (*p <* 0.05); moreover, CS-VAP4/6 had higher expression levels of TLR4 during the period (*p <* 0.05). The expression of TLR6 and TLR9 genes in treated groups were significantly increased compared with CS-pVAX1 at 21 and 28 days (*p <* 0.05), and higher expression levels of TLR6 and TLR9 genes were found in CS-VAP4/6 at 28 days compared with CS-VAP (*p <* 0.05) (Figure 8c,d). Additionally, it was remarkable that the gene expressions of TLR1, TLR4, TLR6, and TLR9 genes reached the maximum at 21 days post-inoculation.

#### 3.6.2. Changes of Expression Levels of Antimicrobial Peptide Gene

**Figure 9:**
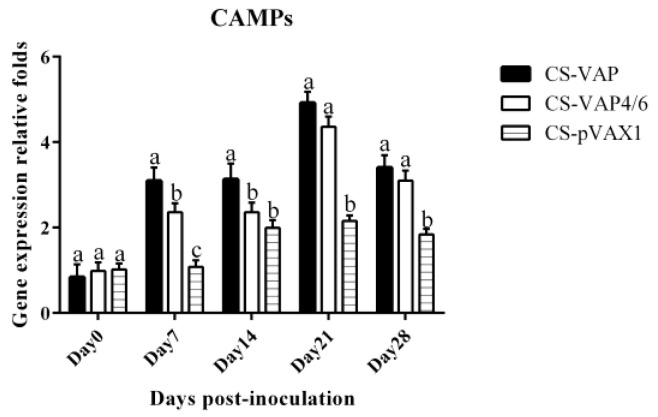
Changes of CAMP gene in blood of experimental mice. It was remarkable that CS-VAP and CS-VAP4/6 got significant increases at 7, 21, and 28 days compared with CS-pVAX1 (*p <* 0.05).

Figure 9 shows the change of the expression of antimicrobial gene CAMP. It was remarkable that CS-VAP and CS-VAP4/6 got significant increases at 7, 21, and 28 days compared with CS- pVAX1 (*p <* 0.05). Meanwhile, the gene expression of all groups reached the highest point at 21 days.

#### 3.6.3. Changes of Expression Levels of Immune Memory Relative Genes 

**Figure 10:**
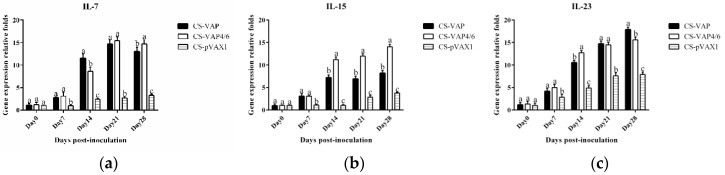
Expression levels of IL-7 (**a**), IL-15 (**b**), and IL-23 (**c**) immune memory genes of experimental mice. The expression levels of IL-7, IL-15, and IL-23 genes in treated groups had significant increases compared with the control group in the whole period (*p <* 0.05). The group treated with CS-VAP4/6 got a higher expression level of IL-15 gene at 14 to 28 days in comparison with CS-VAP (*p <* 0.05). Meanwhile, a higher expression of IL-23 gene was found in CS-VAP4/6 at 14 days compared with CS-VAP (*p <* 0.05).

IL-7, IL-15, and IL-23 were assayed by RT-qPCR (Figure 10a–c) (Figure S1). Expression levels of all three genes were significantly increased compared with controls (*p <* 0.05). From day 7 to day 14, the expression levels of all genes increased dramatically, maximum levels being reached at day 21 or day 28. Expression level of IL-15 was higher in the CS-VAP group than in other groups (*p <* 0.05) but there was no significant difference in levels of IL-7 and IL-23.

#### 3.6.4. Change of Expression Levels of Cytokine Genes

**Figure 11:**
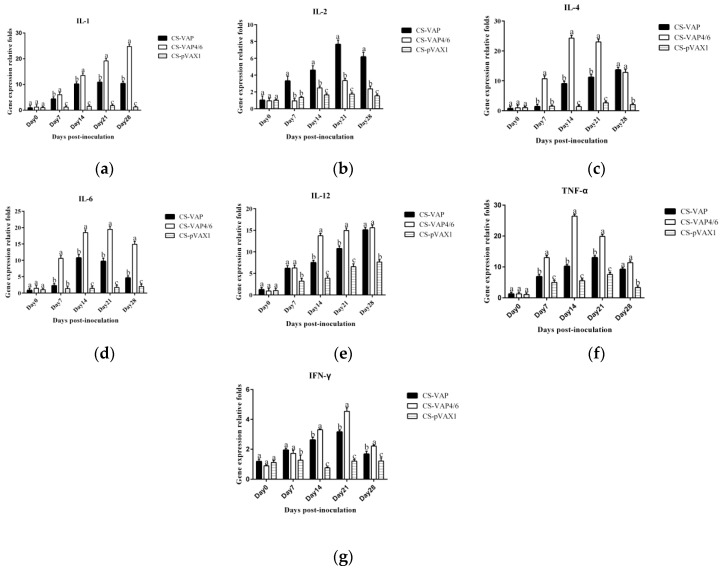
The expression levels of IL-1 (**a**), IL-2 (**b**), IL-4 (**c**), IL-6 (**d**), IL-12 (**e**), TNF-α (**f**) and IFN-γ (**g**) genes. All the gene expression levels of treated groups were significantly increased compared with the control group (*p <* 0.05). CS-VAP4/6 had higher expression levels of IL-1, IL-6, and TNF-α from 7 to 28 days (*p <* 0.05). Additionally, the group treated with CS-VAP4/6 got higher expression levels of IL-4, IL-12, and IFN-γ genes at 14 and 21 days in comparison with CS-VAP (*p <* 0.05).

In order to evaluate the immune response of experimental mice, the gene expression levels of IL-1, IL-2, IL-4, IL-6, IL-12, IFN-γ, and TNF-α were detected. The results are shown in Figure 11. Figure 11a,d,f show that there were significant increases in expression levels of IL-1, IL-6, and TNF-α of CS-VAP and CS-VAP4/6 compared with CS-VAP from 7 to 28 days (*p <* 0.05), respectively; moreover, CS-VAP4/6 had higher expression levels of IL-1, IL-6, and TNF-α during such a period (*p <* 0.05). Expression levels of IL-4, IL-12, and IFN-γ genes were markedly up-regulated in groups CS-VAP and CS-VAP4/6 from 7 to 28 days compared with CS-pVAX1 (*p <* 0.05); meanwhile, the group treated with CS-VAP4/6 got higher expression levels of IL-4, IL-12, and IFN-γ genes at 14 and 21 days in comparison with CS-VAP (*p <* 0.05) (Figure 11c,e,g). Figure 11b shows that the expression levels of IL-2 gene in groups CS-VAP and CS-VAP4/6 were significantly increased compared with CS-pVAX1 at 14, 21, and 28 days (*p <* 0.05), and a higher expression level of IL-2 gene was found in CS-VAP at the same time compared with CS-VAP4/6 (*p <* 0.05). It was remarkable that most of the genes reached the highest expression level at 21 days post-inoculation.

### 3.7. Responses to Challenge

**Figure 12:**
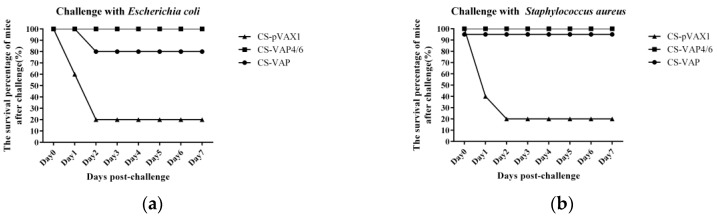
The survival percentage of mice after challenge. It was notable that the mice treated with CS-VAP4/6 respectively showed 100% protection rate against *E. coli* (**a**) and *S. aureus* (**b**). Moreover, the group treated with CS-VAP showed a protection rate of 80% and 100% against *E. coli* and *S. aureus*, respectively. Meanwhile, the control group only had 20% survival percentage.

The data of the survival percentage of mice after challenged intraperitoneally with tolerance bacteria at 28 days post-inoculation are shown in Figure 12, according to the result of our observation and recording every 24 h until 7 days after challenge. The group treated with CS-VAP4/6 demonstrated the best protection against both bacteria, which exhibited 100% survival percentage, respectively. The group treated with CS-VAP only showed 20% mortality after the challenge by *E. coli*. Furthermore, 80% of the mice in group CS-pVAX1 were dead after challenge with two bacteria. In contrast, the survival in the CS-VAP challenged with *E. coli* was 80% of the CS-VAP4/6 group (Figure 12a), and all mice of the treated groups survived after challenge with *S. aureus*.

Subsequent pathological examination indicated that all the dead mice displayed severe lesions, including spleen swelling and bleeding, liver necrosis, lymph nodes bleeding, diffuse bleeding in the stomach, duodenum, and jejunum catarrh. However, the organs and tissues of survived mice displayed few lesions.

## 4. Discussion

The crisis of resistance is a continuously growing problem due to abusing antibiotics, so there is a pressing need for the replacement of antibiotics. AMPs, as essential components of a multicellular immune system, are currently used in development of an anti-infective drug. Studies show that AMPs exhibit pharmacodynamic properties, which will prevent the evolution of resistance in target microbes [18]. With regard to animal immune modulator, cytokines are being intensively studied due to their complicated and vital effects on immune response [19]; for instance, IL-4 and IL-6 can enhance the development of Th2 cells and antibody production, which are necessary for defense against extracellular pathogens [20]. Combing the advantages of these, we wanted to explore whether AMPs and cytokines can synergistically enhance the immune response and anti-infection of mice.

In this study, recombinant plasmids VAP and VAP4/6 were constructed for the first time and encapsulated with chitosan nanoparticles. We then assessed the immunological changes induced by the expression of VAP and VAP4/6 in vivo. In our experiment, the special 2A sequence was used to link CAMPs and IL-4/6 to produce two fusion proteins. This rational molecular design guarantees the bioactivity of CAMPs and IL-4/6, which was confirmed by the in vitro lymphocytes proliferation. There is no significant difference of CAMPs expression between CS-VAP and CS-VAP4/6 on 0, 21, and 28 days. However, on 7 and 14 days, CS-VAP had a higher expression level of CAMPs due to its possible more gene copies.

The TLRs, a family member of pattern-recognition receptors (PRRs), are transmembrane proteins that have specialized recognition of bacteria. Through pattern-recognition receptors (PRRs), innate immune cells are able to detect microbial pathogens. TLR1, TLR4, and TLR6 primarily recognize molecules unique to bacteria, and thus, allow for efficient discrimination from non-self [21]. TLR9 is typically considered as a sensor for CpG DNA, which is relevant to viral and bacterial infections [22]. Interestingly, a large number of AMPs have been found to induce TLR9 signaling through AMP-dsDNA complexes [23,24]. We observed up-regulations of TLR1, TLR4, TLR6, and TLR9 in CS-VAP and CS-VAP4/6 groups, implying the enhanced recognition of pathogens, which also coincided with increased IL-1, IL-2, IL-12, IFN-γ, and TNF-α. IL-1 is a pro-inflammatory factor that promotes antibody production and induces IL-2 production through T-cell helper cells. IL-2, IL-12, IFN-γ, and TNF-α can promote Th1 cell-mediated immune responses to target intracellular pathogens. The changes of cytokine levels are generally correlated with the increase of CD4+T and CD8+T cells. In Figure 11, especially on 14, 21, and 28 days dpi, the increment of IL-1, IL-4, IL-6, IL-12, TNF-α, and IFN-γ ordinarily match up with the increase of CD4+ and CD8+ T lymphocytes in Figure 6a,b, except for 7 dpi in Figure 6c. The possible reason may be the diversified resource of cytokines from different immune cells. Other immune cells besides T lymphocytes, such as macrophages and dendritic cells, also produced cytokines into blood upon stimulation, which can affect the changes of cytokines in the sera.

Moreover, cytokines IL-7 and IL-15 are able to regulate the pool of memory T cells corporately through low-level homeostatic division. IL-7 is primarily important for survival of memory CD8+ cells while IL-15 is vital for low-level proliferation to maintain the size of memory T-cells pool. IL-23 is best known for its ability to promote Th17 maturation. The raised expressions of IL-7, IL-15, and IL-23 in VAP and VAP4/6 mice indicated the up-regulation of the immune memory, which is consistent with the amplification of cellular immunity resulting from the addition of CD4+ and CD8+ T cells.

The result of flow cytometry showed that compared with the control mice, CD4+ and CD8+ T lymphocytes significantly increased in the mice treated with CS-VAP4/6 and CS-VAP, and the CS-VAP4/6-treated mice showed a higher increase than the CS-VAP group, indicating that the humoral immunity of treated mice was efficiently elevated, which was confirmed by the increased IgG, IgG1, and IgG2a in the sera. The increase of IgG was initially stimulated by the inoculation of recombinant plasmids and the expression of inserted immune genes, and it also possibly declined with the degrade of the recombinant plasmids in vivo after 7 dpi. To our knowledge, the mixed inoculation of CS-plasmid was responsible for the IGs increment in the control mice post-day 0, which probably resulted from the stimulation of specific immunity by CS-pVAX1 to the conditioned pathogen in the control mice. These results were in accordance with the reports that the expression of IL-4 gene significantly increased the quantity of IgG1- [25,26] and IL-6- stimulated CD4+ T cells to promote the production of IgG [26].

The challenge results of both *E. coli* and *S. aureus* separately showed the strong protective effect of the recombinant plasmid nanoparticles. Most of the treated mice survived and did not show any lesions or symptoms caused by the infection of bacteria. In contrast, the control mice were severely infected by the challenge bacteria, and most of them died of infection lesions and damage within 48 h. Additionally, there was no death in the next 5 days, so we stopped the observation on 7 days post-challenge due to limited time for experiment in our lab. Among the treated groups, CS-VAP4/6 manifested better improvement of innate and adaptive immunity (*p <* 0.05) and resulted in stronger resistance against challenge with bacteria, which is in accordance with the higher level of humoral and cellular immune responses. Similarly, the CS-VAP4/6 inoculation provoked more systemic immunity in the blood of mice compared with CS-VAP.

Chitosan was utilized as the DNA delivery material, not only for high transfection efficiency but also for its safety and compatibility in vivo [27,28]. So, we selected chitosan to entrap the recombinant plasmids and charge suitable nanoparticles with positive potentials, which can protect the plasmids from digestion. The properties of encapsulated nanoparticles showed that they can effectively transport the DNA to target cells. The proliferation of pig lymphocytes manifested the stimulatory effect of nanoparticles, indicating that the nanoparticles can express bioactive interleukins and CAMPs proteins, which can stimulate lymphocyte proliferation of pigs. It is noteworthy that the stimulatory effect of CS-VAP4/6 was stronger than those of CS-VAP and CS-pVAX1, demonstrating that the fusion gene has better immunological bioactivity. The enhanced expressions of cytokine genes demonstrated that the recombinant plasmid nanoparticles can not only improve the systemic immunity but also slows the process of releasing. The latter was predictable due to the chitosan function of long-term release [29]. It was notable that the expression levels of most of the immune genes reached the highest point at 21 days post-inoculation, and it was significantly upgraded at day 28 post-inoculation, indicating that the nanoparticles have the effect of producing reliable releasing for nearly one month.

Here, the recombinant plasmid of co-expression of CAMPs and IL-4/6 was firstly constructed and entrapped with chitosan nanoparticles, which is used as a potential replacement of antibiot Many indicators consolidated that both of innate and adaptive immunity were enhanced, and the immunity against bacterial infection was also elevated for one month or so, which probably could not protect the mice in a medium- or long-term due to the limited existence of recombinant plasmid in vivo.

## 5. Conclusions

These suggest that the co-expression of fusion antimicrobial peptide gene with interleukin genes could promote better innate and adaptive immunity of animals, and result in a stronger immune defense against pathogenic infection, which would inspire further development of novel and economical practicable immune modulators for the control and prevention of animal diseases in the future.

## Figures and Tables

**Table 1:** The information of four antimicrobial peptides.

Name	Kinds	Spectrum	Mature Sequences
PR-39	Cathelicidin	G−	RRRPRPPYLPRPRPPPFFPPRLPPRIPPGFPPRFPPRFPGKR
Tritrpticin	Cathelicidin	G+, G−	VRRFPWWWPFLRR
PAMP-23	Cathelicidin	G+, G−	RIIDLLWRVRRPQKPKFVTVWVR
PG-1	Cathelicidin	G+, G−, F, V	RGGRLCYCRRRFCVCVGRG

G+: Gram-positive bacteria, G−: Gram-negative bacteria, F: Fungus, V: Virus.

**Table 2:** The primers for RT-PCR.

Gene	Oligonucleotide Sequence (5′-3′)
β-actin-F	TACGCCAACACGGTGCTGTC
β-actin-R	GTACTCCTGCTTGCTGATCCACAT
TLR-1-F	GGACCTACCCTTGCAAACAA
TLR-1-R	GGTGGCACAAGATCACCTTT
TLR-4-F	ACCTGGCTGGTTTACACGTC
TLR-4-R	CTGCCAGAGACATTGCAGAA
TLR-6-F	CCAAGAACAAAAGCCCTGAG
TLR-6-R	TGTTTTGCAACCGATTGTGT
TLR-9-F	ACTGAGCACCCCTGCTTCTA
TLR-9-R	AGATTAGTCAGCGGCAGGAA
IL-1-F	TGCTGTCGGACCCAT
IL-1-R	TGTGCCGTCTTTCATTAC
IL-2-F	AAGCACAGCAGCAGCAGCAG
IL-2-R	GCCGCAGAGGTCCAAGTTCATC
IL-4-F	GCCATATCCACGGATGCGACAA
IL-4-R	GGTGTTCTTCGTTGCTGTGAGGA
IL-6-F	TCTTGGGACTGATGCTGGTGACA
IL-6-R	AGCCTCCGACTTGTGAAGTGGTAT
IL-7-F	TTCCTCCACTGATCCTTGTTCT
IL-7-R	AGCAGCTTCCTTTGTATCATCAC
IL-12-F	CAATCACGCTACCTCCTCTTTT
IL-12-R	CAGCAGTGCAGGAATAATGTTTC
IL-15-F	CATCCATCTCGTGCTACTTGTG
IL-15-R	GCCTCTGTTTTAGGGAGACCT
IL-23-F	TGCTGGATTGCAGAGCAGTAA
IL-23-R	GCATGCAGAGATTCCGAGAGA
IFN-γ-F	AGGCCATCAGCAACAACATA
IFN-γ-R	TGAGCTCATTGAATGCTTGG
TNF-α-F	CCTGTAGCCCACGTCGTAG
TNF-α-R	GGGAGTAGACAAGGTACAACCC
CAMP-F	CAGCAGTCCCTAGACACCAAT
CAMP-R	CACAGACTTGGGAGTATCTGGA

F: forward, R: reverse.

## Supplementary Materials

The following are available online at https://www.mdpi.com/2076-393X/8/3/552/s1, Figure S1: The raw data of gene expressions of IL-7, IL-15 and IL-23 by QPCR.
